# ﻿*Decussiphycussinensis* sp. nov. (Bacillariophyceae, Mastogloiales) – a new species described from China, with comments on phylogenetic position of the genus

**DOI:** 10.3897/phytokeys.254.142654

**Published:** 2025-03-18

**Authors:** Andrei Mironov, Anton Glushchenko, Elena Kezlya, Yevhen Maltsev, Anton Iurmanov, Yan Liu, Maxim Kulikovskiy

**Affiliations:** 1 College of Life Science and Technology, Harbin Normal University, Harbin, 150080, Heilongjiang Province, China Harbin Normal University Harbin China; 2 К.А. Timiryazev Institute of Plant Physiology RAS, IPP RAS, 35 Botanicheskaya St., Moscow, 127276, Russia К.А. Timiryazev Institute of Plant Physiology RAS, IPP RAS Moscow Russia; 3 Faculty of Biology, M.V. Lomonosov Moscow State University, Leninskie Gory 1, building 12, Moscow, 119234, Russia M.V. Lomonosov Moscow State University Moscow Russia

**Keywords:** Bacillariophyceae, *Decussiphycus*, Hainan Province, new species, phylogeny, pore occlusions

## Abstract

During the study of freshwater diatom communities in Hainan Province, China, we uncovered an unknown diatom species of the genus *Decussiphycus*, which is described as *Decussiphycussinensis***sp. nov.** herein. The description is based on LM and SEM investigations; morphologically, the new species is compared to other taxa belonging to the genus. We complemented the description with the results of a molecular analysis based on SSU rDNA and *rbc*L sequencing. Molecular data is acquired for *Decussiphycus* for the first time. Hereby, we discuss the phylogenetic relationships between this genus and its closest allies – *Aneumastus* and *Mastogloia*, demonstrating the affinity of *Decussiphycus* within the order Mastogloiales.

## ﻿Introduction

Throughout the years, diatomists praised different kinds of evidence while describing new species and genera or analyzing the taxonomy and phylogeny of high-rank groups (i.e. families and orders). While some taxonomists valued chloroplast morphology (Mereschkowsky 1903; Heinzerling 1908; Cox 1987, 2015), others appraised valve features (Cleve 1891; Hustedt 1930; Mann 1984; Kociolek and Stoermer 1988; Krammer 2002). However, diatom taxonomy has nowadays shifted towards the adoption of a “narrow” species concept (Mann 1999). Most of all, this trend influenced the taxonomic revision of the “catch-all” genera, e.g., *Navicula* Bory. Several genera, for example *Placoneis* Mereschkowsky and *Geissleria* Lange-Bertalot & Metzeltin were separated from *Navicula* after re-examinations of chloroplast and valve morphology (Mereschkowsky 1903; Lange-Bertalot and Metzeltin 1996). Another genus introduced in this way is *Decussata* (R.M. Patrick) Lange-Bertalot *nom. inval.*, which has been recently renamed (Guiry and Gandhi 2019) as *Decussiphycus* Guiry & Gandhi in accordance with Art. 20.2 of the ICN (Shenzhen Code; Turland et al. 2018). Similarly, the mentioned nomenclature alteration was applied by Wynne (2019) to replace *Delicata* Krammer *nom. inval.* with *Delicatophycus* M.J. Wynne. Lately, this approach was criticized by da Silva (2024) and thus, the validity of the names *Delicatophycus* and *Decussiphycus* remains unclear until the publication of the Madrid edition of the International Code of Nomenclature for Algae, Fungi, and Plants.

Originally, *Decussiphycus* emerged from *Naviculaplacenta*-group (Patrick 1959) and was later granted with a status of an independent genus by Lange-Bertalot (2000). Edlund et al. (2006) emended the description of *Decussiphycus* (at the time – *Decussata nom. inval.*), focusing on both chloroplast and valve characters to distinguish it from other genera. According to their diagnosis, *Decussiphycus* includes diatoms with two chloroplasts of complex configuration (H-shaped in girdle view, each with four apically elongated lobes) near each pole. The valves are flat, rectangular in girdle view, equipped with a narrow mantle, circular central area, filiform raphe, crozier-shaped proximal raphe ends and distal ends deflected in opposite directions. The most notable feature of the genus is decussate or quincunx arrangement of striae (Edlund et al. 2006). Several ultrastructural features of *Decussiphycus* should be listed as well: slight sinuous discontinuity of the raphe near the apices, perforated bands of cingulum (each with two rows of poroids), areolae occluded by “circular convex hymene” (sensu Edlund et al. 2006).

After its emergence at the genus-level, *Decussiphycus* has been considered to represent the order Mastogloiales D.G. Mann, which has been originally made up of *Aneumastus* D.G. Mann & Stickle and *Mastogloia* Thwaites ex W. Smith, solely (Round et al. 1990). Subsequently, Cox (2015) elaborated a new concept of the order by emending the descriptions of Achnanthaceae Kützing and Mastogloiaceae Mereschkowsky. Therefore, Mastogloiales was supplemented with a monoraphid genus *Craspedostauros* Cox. Cox’s system implied that the synapomorphies of *Craspedostauros* and biraphid mastogloioid diatoms are cribrate areolae and presence of two H-shaped chloroplasts. However, the relevance of Cox’s proposals is still being contested. Similarly, until today, the accurate phylogenetic position of the genus *Decussiphycus* has been doubtful due to the complex morphology of the genus (i.e. intricate structure of pore occlusions) and, most importantly, the lack of molecular data. In this paper, we provide the results of a new molecular analysis based on SSU rDNA and *rbc*L sequencing, demonstrating the phylogenetic position of *Decussiphycus* for the first time defining its position within the order Mastogloiales.

Hitherto, the genus *Decussiphycus* has been comprised by only three morphologically close taxa – *Decussiphycusplacenta* (Ehrenberg) Guiry & Gandhi, Decussiphycusplacentavar.obtusus (F. Meister) Guiry & Gandhi and *Decussiphycushexagonus* (Torka) Guiry & Gandhi. One unknown *Decussiphycus* species was found during the survey in the area of Wuzhishan Mountain in the Province of Hainan, China. Notably, as multiple studies revealed (Kociolek et al. 2015; Kulikovskiy et al. 2015, 2018; Glushchenko and Kulikovskiy 2017; Glushchenko et al. 2017a, 2019, 2020; Liu et al. 2018; Maltsev et al. 2019; Mironov et al. 2024), diatom communities in the region of South China and surrounding territories are characterized by considerable level of diversity. Several species of the order Mastogloiales, e.g. *Aneumastuslaosica* Glushchenko, Kulikovskiy & Kociolek and *Aneumastusgenkalii* Glushchenko, Kulikovskiy & Kociolek, were described from this area (Glushchenko et al. 2017b), too. In this study, based on unique combination of valve features and molecular data, we describe one more species of this order – *Decussiphycussinensis* Glushchenko, Maltsev, Mironov, Liu & Kulikovskiy sp. nov.

## ﻿Methods

### ﻿Sample collection and preparation

In the current study, we investigated a single sample of diatom biofilms, collected from an unnamed mountain stream at the slope of Wuzhishan Mountain, Hainan Province, China. The sample was treated with 10% hydrochloric acid to remove carbonates and then washed with deionized water for 12 h. To remove the organic matter, boiling in concentrated hydrogen peroxide (37%) was applied. Furthermore, the sample was washed with deionized water four times with 12 h intervals. It was then decanted and filled with deionized water up to 100 ml; the suspension was pipetted onto coverslips. Afterwards, it was left for drying at room temperature. A permanent sample was mounted in Naphrax® (refractive index = 1.73). Live material was viewed with a Zeiss Axio Scope A1 microscope with mounted Axiocam ERc 5s camera (Zeiss, Germany) and equipped with an oil immersion EC Plan-NEOFLUAR objective (x100, n.a. 1.3) for epifluorescent microscopy (EFM) and an oil immersion Plan-apochromatic objective (x100, n.a. 1.4; Nomarski differential interference contrast) for LM of cleaned material.

Later, a part of the suspension was spread onto aluminum stubs after air-drying at room temperature for 24 h in order to prepare SEM stubs. The stubs were then sputter-coated with 50 nm of Au by the means of Eiko IB 3 apparatus (Eiko Engineering, Japan). For SEM investigations, we applied the TESCAN Vega III (TESCAN, Brno, Czech Republic) in the Borissiak Paleontological Institute of the Russian Academy of Science. The suspension and slides analyzed herein are deposited in the collection of Maxim Kulikovskiy at the Herbarium of the Institute of Plant Physiology Russian Academy of Sciences, Moscow, Russia.

The terminology of the valve follows Lange-Bertalot (2001), Edlund et al. (2006), Stancheva and Temniskova (2006), Kulikovskiy et al. (2016).

### ﻿Culturing and DNA preparation

The monoclonal strain Ca68 was established by micropipetting a single cell under a Zeiss Axio Vert. A1 inverted microscope (with × 10 objective). The strain was cultivated in WC liquid medium (Guillard and Lorenzen 1972) in Petri dishes at 23 °C with an alternating 12-hour light and dark photoperiod.

Genomic DNA was extracted with Chelex100 Chelating Resin (Bio-Rad Laboratories, Hercules, CA, USA) with primers D512for and D978rev for SSU rDNA (Zimmermann et al. 2011); dp7- (Daugbjerg and Andersen 1997) and *rbc*L404+ (Ruck and Theriot 2011) for *rbc*L. ScreenMix (Evrogen, Moscow, Russia) was utilized for the PCR. Amplified material was visualized by horizontal electrophoresis in agarose gel (1.0%) stained by the SYBR^TM^ Safe (Life Technologies, Carlsbad, CA, USA). Sequencing procedure was conducted with a Genetic Analyzer 3500 instrument (Applied Biosystems, Waltham, MA, USA).

### ﻿Molecular analysis

Molecular analysis, as performed in this study, follows the algorithms described in Mironov et al. (2024) and Tseplik et al. (2024).

**Figure 1.**
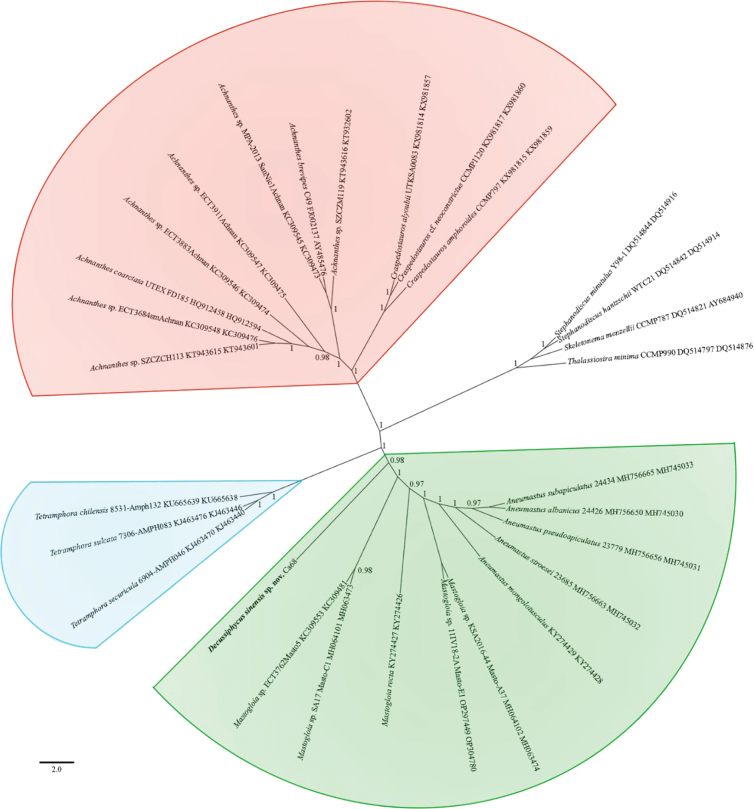
Phylogenetic position of *Aneumastus*, *Mastogloia* and *Decussiphycus* species based on BI from an alignment of 29 sequences and 1,353 characters (*rbc*L and SSU rRNA genes). Values of PP below 0.9 are hidden. Strain numbers (if available) and GenBank numbers are indicated for all sequences.

The dataset for multigene analysis was comprised of 29 concatenated SSU rDNA and *rbc*L sequences, selected for available lineages of 25 representatives of Mastogloiales sensu Cox (2015) and four diatom species from Thalassiosirophycidae Round & R.M. Crawford chosen as the outgroups (taxa names and Accession Numbers are given in Fig. 1). The SSU rDNA and *rbc*L sequences were aligned in separately by the means of the G-INS-I algorithm using the Mafft ver. 7 software (RIMD, Osaka, Japan) (Katoh and Toh 2010). The dataset used in further analysis included 1,795 and 1,493 nucleotide sites for nuclear SSU rDNA, and plastid *rbc*L regions, respectively. After that, unpaired regions were eliminated, and the resulting aligned SSU rDNA sequences were combined with the *rbc*L sequences into a united matrix for concatenated SSU rDNA and *rbc*L. Alignments used for phylogenetic analyses are presented in supplementary files (Suppl. material 1).

The Bayesian inference (BI) method was conducted with Beast ver. 1.10.1 software (BEAST Developers, Auckland, New Zealand) (Drummond and Rambaut 2007). Most suitable partition-specific substitution models, shape parameter α and a proportion of invariable sites (pinvar) were found out with the help of the Bayesian information criterion (BIC) in jModelTest ver. 2.1.10 software (Vigo, Spain) (Darriba et al. 2012). During the BIC-based model selection procedure, we chose the following models, shape parameter α and a proportion of invariable sites (pinvar): GTR+G+I, α = 0.4710 and pinvar = 0.5970 for SSU rDNA; TPM1uf+G+I, α = 0.3960, and pinvar = 0.7310 for the first codon position of the *rbc*L gene; JC+I, pinvar = 0.8690 for the second codon position of the *rbc*L gene; GTR+G+I, α = 1.1260, and pinvar = 0.2320 for the third codon position of the *rbc*L gene. Besides, the HKY and F81 models were applied instead of TPM1uf and JC, respectively, as the most similar suitable options for BI. Speciation procedure was performed by a Yule process tree prior. Five MCMC analyses were conducted for 5 million generations (burn-in 1,000 million generations). Tracer ver. 1.7.1 software (MCMC Trace Analysis Tool, Edinburgh, United Kingdom) (Drummond and Rambaut 2007) was utilized for the convergence diagnostics. Furthermore, the initial 15% trees were eliminated, while the rest were retained for final chronogram construction (with 90% Bayesian posterior probabilities – PP). The Bayesian phylogenetic topology for the *rbc*L and SSU rRNA genes tree is attached as a supplementary file (Suppl. material 2). Phylograms were viewed and edited with FigTree ver. 1.4.4 (University of Edinburgh, Edinburgh, United Kingdom) and Adobe Photoshop CC ver. 19.0 software.

## ﻿Results

### ﻿Molecular phylogeny of *Decussiphycus*

Phylogeny of the Mastogloiales sensu Cox (2015), based on SSU rDNA and *rbc*L sequencing, is demonstrated in Fig. 1. As illustrated, three genera of the order – *Aneumastus*, *Mastogloia* and *Decussiphycus* comprise an independent monophyletic group (clade *AMD*, highlighted in green), which is highly statistically supported (posterior probability, PP = 0.98). In our molecular analysis, the genus *Decussiphycus* was represented by a single newly acquired strain *Decussiphycussinensis* sp. nov., which is positioned in a separate node, as a basal taxon within the group. The genus *Aneumastus* is demonstrated as monophyletic in the phylogram, with maximum statistical support. On the contrary, molecular data reveals the paraphyly of the genus *Mastogloia*, confirming the assumptions of Kezlya et al. (2024). This revelation, perhaps, indicates the necessity of further re-evaluation of *Mastogloia*. Another independent clade on the phylogram (highlighted in blue) corresponds to the genus *Tetramphora* Mereschkowsky, including 3 strains. The relationship between these clades is also strongly supported (PP = 1.0). The clade comprising *Achnanthes* and *Craspedostauros* (highlighted in red) is isolated from the *AMD* clade the most. That clade, in turn, is also supported with maximum rate.

### ﻿Species description

#### ﻿ Decussiphycus sinensis

Taxon classificationPlantaeMastogloialesMastogloiaceae

Glushchenko, Maltsev, Mironov, Liu & Kulikovskiy sp. nov.

EFC58AC6-829A-5A23-9958-439EEDA6E188

**Figure 2.**
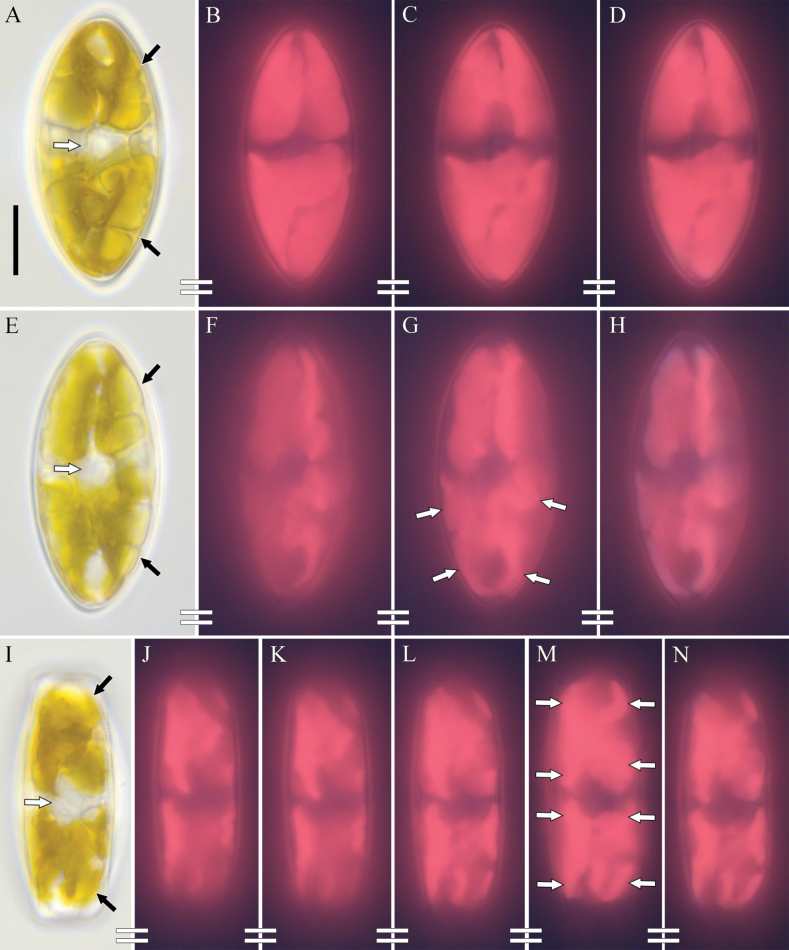
**A–N***Decussiphycussinensis* sp. nov. LM, DIC. Strain Ca68 **A–H** valve face view **A–H** girdle view **A, E, I** live cells. White arrows point to the nucleus, black arrows show the two apically located plastids **B–D, F–H, J–N** chloroplast autofluorescence. White arrows show the four lobes of each plastid. Scale bar: 10 µm.

**Figure 3.**
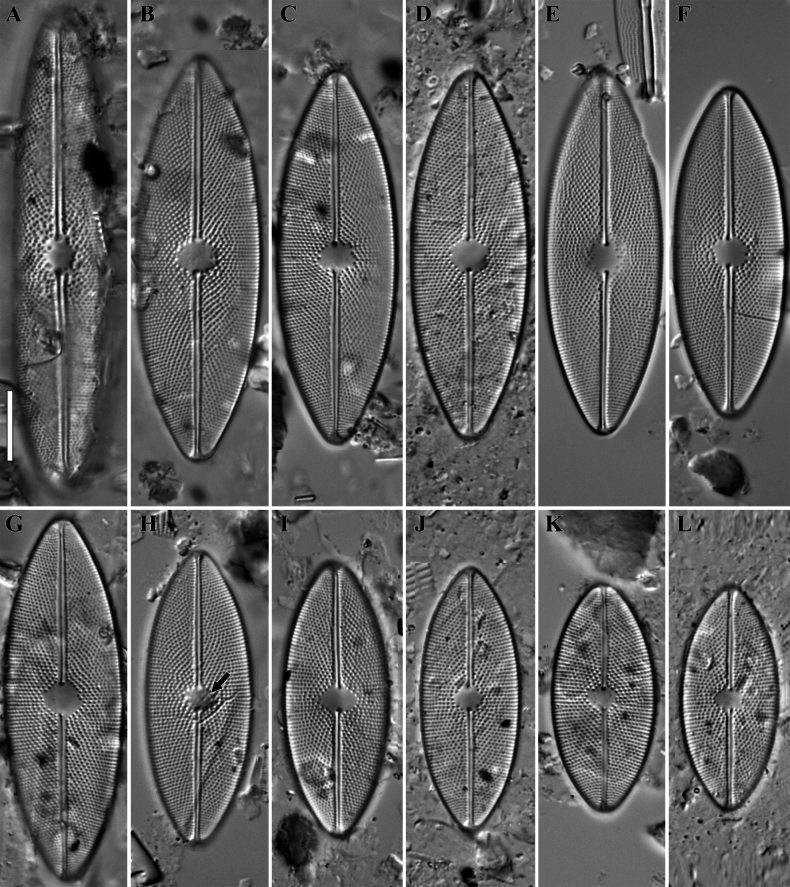
**A–L***Decussiphycussinensis* sp. nov. LM, DIC. Size diminution series. Slide 08909 (from sample ЕHHN 2014043). Post-initial valve (**A**). Black arrow shows the randomly located areolae (**H**). Scale bar: 10 µm.

**Figure 4.**
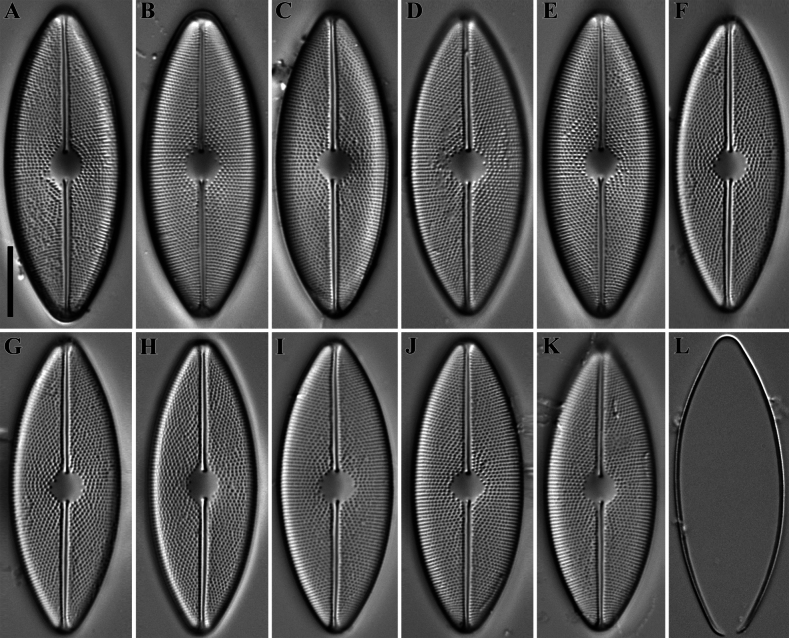
**A–L***Decussiphycussinensis* sp. nov. LM, DIC. Slide 09153 (from strain Ca68). Holotype (**C**) A residual cingulum separated from the valve (**L**). Scale bar: 10 µm.

**Figure 5.**
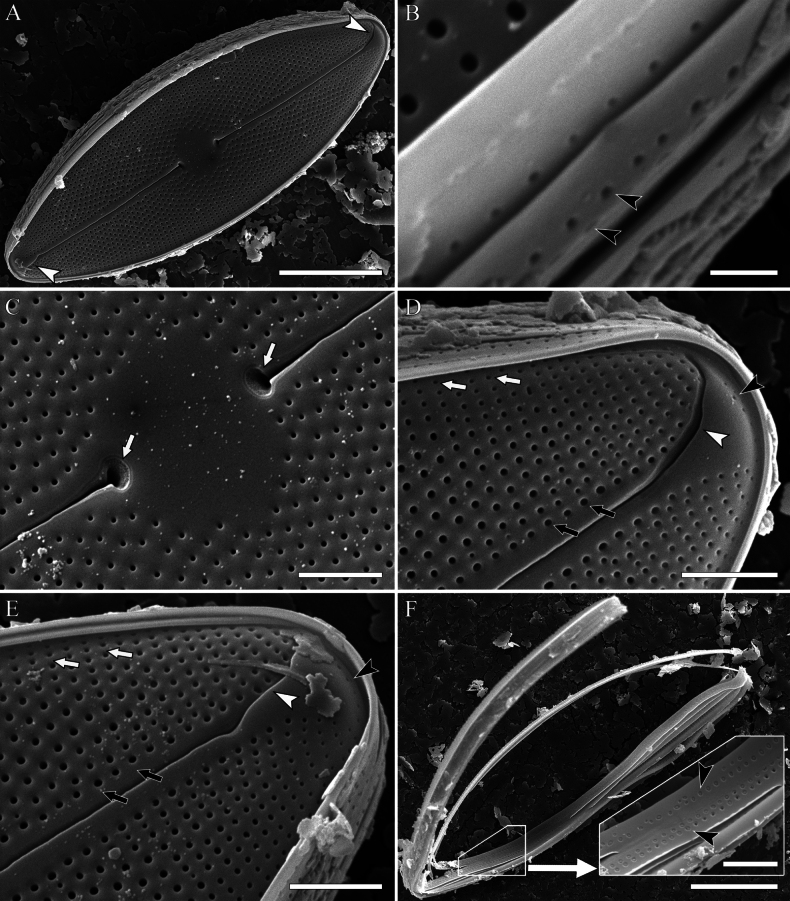
**A–F***Decussiphycussinensis* sp. nov. SEM, external view. Slide 09153 (from strain Ca68) **A** the entire valve; note the oppositely deflected distal raphe fissures **B** details of girdle bands structure; note the two rows of areolae (black arrowheads) **C** details of the central area; proximal raphe fissures expanded, slightly tilted to one side (white arrows) **D, E** details of the valve apex; note the distal raphe ends with silica folds (white arrowheads), areolae larger near the axial area (black arrows) and smaller towards the margin (white arrows), an isolated row of areolae (black arrowheads) **F** girdle bands; note the double rows of areolae with silica caps (black arrowheads). Scale bars: 10 µm (**A, F**); 0.5 µm (**B**); 2 µm (**C–E**).

**Figure 6.**
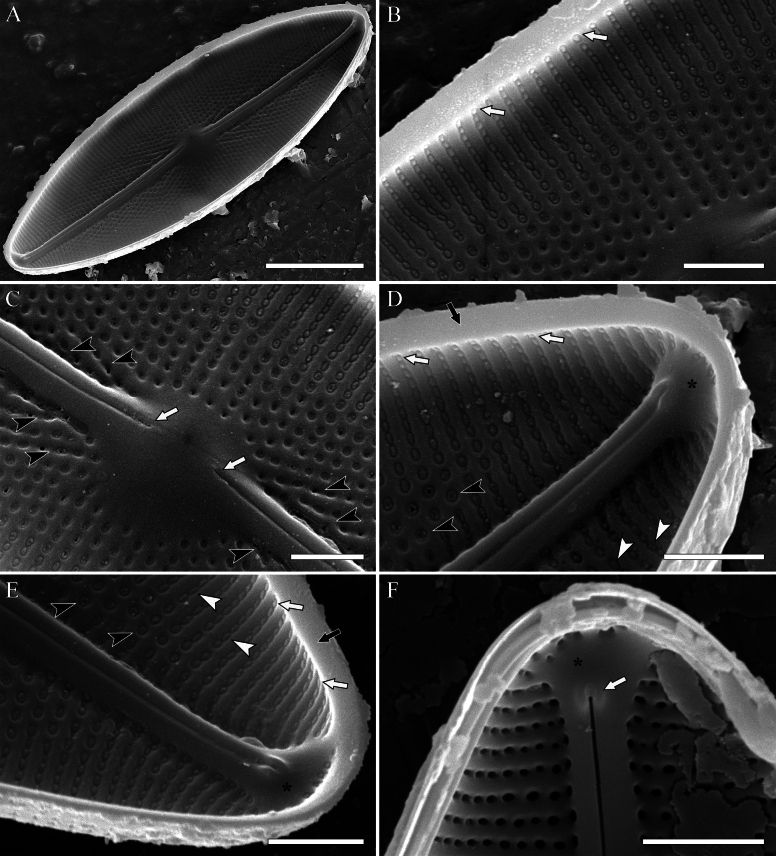
**A–F***Decussiphycussinensis* sp. nov. SEM, internal view. Slide 09153 (from strain Ca68) **A** the entire valve **B** details of striae structure near the valve margin; note the shallow margin (white arrows) **C** details of the central area; note proximal raphe fissures (white arrows) and ribs of the quincunx system (black arrowheads) **D, E** details of the valve apex; note the shallow valve margin (black arrows) with striae continuing onto it (white arrows), areolae with round occlusions near the sternum (black arrowheads) and oval occlusions towards the margin (white arrowheads). Apical expansions of the sternum indicated by asterisks **F** details of the valve apex; note the horseshoe-shaped helictoglossa (white arrow) and apical expansion of the sternum (asterisk). Scale bars: 10 µm (**A**); 2 µm (**B–F**).

Figs 2 , 3 , 4 , 5 , 6

##### Holotype.

Slide 09153 in herbarium of MHA, Main Botanical Garden, Russian Academy of Science, Moscow, Russia, represented here by Fig. 4C.

##### Isotype.

Slide 08909 in herbarium of MHA, Main Botanical Garden, Russian Academy of Science, Moscow, Russia.

##### Type.

China. Hainan Province, unnamed stream at the northern slope of Wuzhishan Mountain, biofilms on rocks, 18.9815°N, 109.6854°E, 470 m asl, leg. Y. Liu, 12.07.2014. Slide 09153 from oxidized culture strain no. Ca68, isolated from sample THHN 2014043.

##### Representative specimens.

Strain Ca68 (slides 09153); sample THHN 2014043 (slide 08909).

##### Sequence data.

GenBank accession numbers PV016799 (strain Ca68, partial SSU rRNA gene sequence, V4 region); PV021297 (strain Ca68, partial rbcL sequence).

##### Description.

***Live cells*** (Fig. 2A–N). Cells solitary. Nucleus located centrally within a cytoplasmic bridge between the central nodules (Fig. 2A, E, I, white arrows). Each cell contains two chloroplasts of complex configuration, each of which is located at in the apical valve regions (Fig. 2A, E, I, black arrows). In the valve face view, both of the plastids are invaginated along the apical axis to create a central plastid isthmus. In the girdle view, each chloroplast is H-shaped, with four clearly visible arms that extend along the surface of the valve, in its plane (Fig. 2M, white arrows). The arms reach the cingulum region. The four lobes can be discerned in valve view as well (Fig. 2G, white arrows).

**LM** (Figs 3, 4). The post-initial valve has a linear shape with slightly convex margins and bluntly curved ends; length – 77.5 µm, width – 16.9 µm. Distal raphe ends recurved in opposite directions, terminating to the valve face (Fig. 3A). Valves linear–elliptical to elliptical with broadly rounded ends. Length 32.1–69.1 µm, width 13.9–18.3 µm. Axial area narrow, linear. Central area transapically oval to circular. Sometimes, a few randomly located areolae, visible with careful focusing, are positioned at the central area (Fig. 3H, black arrow). Raphe filiform, straight to slightly undulate. Central raphe ends drop-shaped. Distal raphe ends deflected to the valve margin. Striae are decussate, formed by clearly visible areolae forming the right quincunx. Areolae are arranged in multiple rows – a transapical row and two oblique rows which cross each other at angles of 60°–80°. Transapical striae 21–23 in 10 µm. Occasionally, residual cingula can be found separated from the valve (Fig. 4L).

**SEM, *external view*** (Fig. 5A–F). Valve face is flat. Central raphe ends slightly tilted to one side and lie in asymmetrical depressions (Fig. 5C, white arrows). Distal raphe ends oppositely deflected (Fig. 5A, white arrowheads), bordered by small silica folds (Fig. 5D, E, white arrowheads). Areolae small, rounded, their diameters are slightly larger near the axial area (Fig. 5D, E, black arrows) and smaller towards the valve margin (Fig. 5D, E, white arrows). The apex of the valve is equipped with a single isolated row of areolae, situated behind the distal raphe ends (Fig. 5D, E, black arrowheads). Cingulum composed of open 3–4 girdle bands (Fig. 5F). Two rows of areolae are located on each girdle band (Fig. 5B, black arrowheads). Girdle band areolae are smaller than areolae at the valve and arranged alternately or, sometimes, chaotically. Rarely, one of the rows is interrupted. Notably, each areola in the girdle bands is covered with a layer of silica (Fig. 5F, black arrowheads).

**SEM, *internal view*** (Fig. 6A–F). The valve margins is shallow (Fig. 6D, E, black arrows). The striae continue onto the valve margins (Fig. 6B, D, E, white arrows). The interstriae are slightly raised relatively to the striae. Oblique ribs of the quincunx system are located near the central area. They are more elevated in comparison to the transapical interstriae (Fig. 6C, black arrowheads). Areolae vary in size, shape, and type of occlusions. Areolae located closer to the sternum are distinguished by a larger diameter, round shape and presence of flat, slightly depressed, rounded silica formations (Fig. 6D, E, black arrows). Areolae, located near the valve margin are transapically elongated, covered with oval, raised silica caps (“convex hymene”, sensu Edlund et al. 2006) (Fig. 6D, E, white arrows). Transapical areolae density – 18–20 in 10 µm. Raphe slits, straight, lying on the raised sternum. The sternum widens towards the valve apex (Figs 6D–F). Central raphe ends straight, not expanded (Fig. 6C, white arrows). Distal raphe ends terminate with well-expressed, horseshoe-shaped helictoglossae (Fig. 6F, white arrow).

##### Etymology.

The specific epithet refers to the name of the country where this species was discovered.

##### Distribution.

So far, the species is known only from the type locality.

##### Ecology.

The species was located in a mountain stream with temperature of 26.7 °C, pH = 7.64 and conductivity = 60 µS/cm.

##### Comments.

Specimens of *D.sinensis* sp. nov. from wild population were 32.1–69.1 µm long, 13.9–18.3 µm wide, with striae width of 21–23 in 10 µm. Specimens from culture are characterized by smaller valves: 41.5–46.1 µm long, 15.5–17.1 µm wide; striae 22–23 in 10 µm, which corresponds to material from wild population.

### ﻿New combination in the genus *Decussiphycus*

#### ﻿ Decussiphycus obtusus

Taxon classificationPlantaeMastogloialesMastogloiaceae

(F. Meister) Glushchenko, Maltsev, Mironov, Liu & Kulikovskiy, comb. et stat. nov.

2C059DA2-F190-5A57-8FFB-1AF3155A9A2A

##### Basionym.

Naviculaplacentavar.obtusa F. Meister 1932. Kieselalgen aus Asien, p. 37, pl. 13, fig. 99.

##### Synonym.

Decussiphycusplacentavar.obtusus (F. Meister) Guiry & Gandhi, 2019.

## ﻿Discussion

### ﻿Morphological comparison *Decussiphycussinensis* sp. nov. with similar species

*D.sinensis* sp. nov. shares a number of similarities with other representatives of the genus (see Table 1), e.g., shape of the central area, decussate striae arrangement, along with ultrastructural valve features – morphology and location of chloroplasts, structure of girdle bands, areolae occluded by “circular convex hymene” (sensu Edlund et al. 2006). Generally, *D.sinensis* sp. nov. and similar species differ from each other, primarily, by valve outlines and morphology of apices.

**Table 1.** Comparison of morphological features of *D.sinensis* sp. nov. and related species.

	*D.sinensis* sp. nov.	*D.placenta*	*D.hexagona*	*D.obtusus*
Valve shape	linear-elliptic to elliptic	broadly elliptic	linear to linear-elliptic	elliptic
Valve ends	broadly rounded	abruptly protracted, narrowly rostrate to subcapitate	narrowed to a wedge, finally obtusely rounded	broadly rounded
Length, µm	32.1–69.1	35–60	25–44	44–60
Width, µm	13.9–18.3	14–20	9–13	21–25
Transapical striae in 10 µm	21–23	20–25	20–25	20
Axial area	narrow, linear	narrow, linear	narrow, linear	narrow, linear
Central area	transapically oval to circular	rather small, broadly elliptic in outline	transapically elliptic	circular
Shape of areolae near the sternum, internally	distinguished by a larger diameter, rounded, covered with flat, slightly recessed rounded silica plates	rounded, transapically elongated and are covered with raised rounded silica plates	rounded, covered with raised silica caps	n.d.
Shape of areolae near the valve margin, internally	become transapically elongated, covered with oval, raised silica caps	n.d.	n.d.	n.d.
Ecology	confined to lotic ecosystems	confined to lotic ecosystems	confined to lotic ecosystems, found in acidified freshwaters, aerophile	confined to lotic ecosystems
Distribution	Southeast Asia, China, Hainan (type locality)	widely distributed	widely distributed	Southeast Asia, Nepal
References	This study	Lange-Bertalot 2001; Kulikovskiy et al. 2016	Lange-Bertalot, 2000, 2001; Stancheva and Temniskova 2006; Kulikovskiy et al. 2016	Meister 1932

*D.sinensis* sp. nov. resembles *D.placenta* by valve width (13.9–18.3 µm in *D.sinensis* sp. nov. vs. 14–20 µm in *D.placenta*) and striae density (21–23 in 10 µm in *D.sinensis* sp. nov. vs. 20–25 in 10 µm in *D.placenta*). However, *D.sinensis* sp. nov. differs from *D.placenta* by broadly rounded and unprotracted apices, while in *D.placenta* valve apices are distinctly protracted, narrowly rostrate to subcapitate (e.g. Lange-Bertalot 2001: p. 452, Pl. 108, figs 11–13, Pl.109, fig. 4).

Among *D.sinensis* sp. nov. and *D.hexagona*, striae densities are comparable: 21–23 in 10 µm in *D.sinensis* sp. nov. vs. 20–25 in 10 µm in *D.hexagona* (Table 1). Regarding the remaining features of the valve, *D.sinensis* sp. nov. differs from *D.hexagona* most prominently. The valves of *D.sinensis* sp. nov. are linear-elliptic to elliptic, valve outlines convex; the valves of *D.hexagona* are mostly linear or linear-elliptic, with weakly convex to nearly parallel outlines (Table 1). Valve width in *D.sinensis* sp. nov. is 13.9–18.3 µm, which significantly exceeds valve width in *D.hexagona* – 9–13 µm (see Table 1). Central area in *D.sinensis* sp. nov. is transapically oval to round, and transapically elliptic in *D.hexagona* (see Table 1). Finally, the two species differ by the shape of apices: they are broadly rounded in *D.sinensis* sp. nov., but rostrate to bluntly rounded in *D.hexagona* (e.g. Lange-Bertalot 2001: p. 452, Pl. 108, figs 14–17).

Both D.placentavar.obtusus and the newly described species are characterized by narrow axial and circular central areas, as well as broadly rounded valve apices. At the same time, the species obviously differ in valve width (13.9–18.3 µm in *D.sinensis* sp. nov. vs. 21–25 µm in D.placentavar.obtusus) and striae density (21–23 in 10 µm in *D.sinensis* sp. nov. vs. 20 in 10 µm in D.placentavar.obtusus). The other ultrastructural morphological features of D.placentavar.obtusus are not studied yet.

### ﻿On the taxonomy of *Decussiphycusobtusus* comb. et stat. nov.

As illustrated by F. Meister (Meister 1932: taf. 13, fig. 99), the morphology of Naviculaplacentavar.obtusa is clearly different from *Decussiphycusplacenta* according to its current conception. The most prominent difference is expressed in the shape of apices. Therefore, we propose transferring Naviculaplacentavar.obtusa to the genus *Decussiphycus* and endowing it with a new status.

### ﻿On the molecular phylogeny of *Decussiphycus*

As described in the introduction, throughout the history of diatom science, taxonomists gave preferences to various types of evidence for their inquiries: from chloroplast characters, to valve structure, to, as nowadays, molecular data. On this challenging course, several mistakes were made, which, consequently, led to misunderstanding some taxa’s systematics and phylogeny. For instance, E. J. Cox (2015) made an attempt to assess the system of diatoms relying on chloroplast morphology. One of her assumptions revolved around *Achnanthes* Bory. In her study, Cox (1999) presumed the homology between *Achnanthes* and *Mastogloia* based on the similarities in plastid arrangement, structure of areolae (presence of cribrate occlusions) and, partially, presence of stauros. However, further molecular analysis (Ashworth et al. 2017), involving the discussed genera, alongside *Craspedostauros* and *Staurotropis* Paddock, did not prove Cox’s hypothesis. As authors demonstrated, *Achnanthes* and *Craspedostauros* are closely allied, rather than related to *Mastogloia*. The same evidence has been acquired as the result of our molecular investigation. In this study, we supplement the monophyly of Mastogloiales, comprising it of three genera – *Aneumastus*, *Decussiphycus* and *Mastogloia*. In fact, as our analysis demonstrate, genera of Mastogloiales sensu Cox (2015) scatter into three groups: *Craspedostauros*+*Achnanthes* clade, *Tetramphora* clade and *Aneumastus*+*Mastogloia*+*Decussiphycus* (*AMD*) clade. According to molecular data, the latter group must be treated as the natural order Mastogloiales.

At the same time, interrelationships within the discussed *AMD* group are still fully obvious. Taxonomic composition of *Mastlogloia*, which is the most species-rich genus of the *AMD* clade (Loir and Navarino 2013), is of particular interest. *Mastlogloia* includes several species with unique morphological features, i.e. *Mastogloiafimbriata* (T.Brightwell) Grunow lacking external terminal raphe fissures and an apical septum (Pennesi et al. 2016), or *Mastogloiacyclops* Voigt, possessing a distinctive stigma (Stephens and Gibson 1980a). The latter case has been recently investigated by Kezlya et al. (2024) who utilized a combined analysis of valve ultrastructure and molecular data to propose a new genus – *Stigmagloia* Glushchenko, Kezlya, Kapustin & Kulikovskiy. Undoubtedly, further morphological and molecular investigations of different species-groups within *Mastogloia* (Stephens and Gibson 1980a, 1980b), could bring novel insights into the phylogeny of the genus itself, as well as the order Mastogloiales in general.

## ﻿Conclusions

Our research describes a new species, *Decussiphycussinensis* sp. nov., and proposes a new combination – *Decussiphycusobtusus* comb. et stat. nov. The new species description is based on a thorough investigation of valve morphology by means of LM and SEM, supplemented with the results of a two-gene molecular analysis. Thus, the new species can be distinguished by a combination of valve features (i.e. valve outlines, shape of apices, ultrastructure of areolae) and molecular data. In addition, we have made an attempt to investigate the morphological and molecular boundaries of the order Mastogloiales and discuss its relations with genera *Craspedostauros* and *Achnanthes*, underscoring the need for further research in this field.

## Supplementary Material

XML Treatment for Decussiphycus sinensis

XML Treatment for Decussiphycus obtusus

## ﻿Additional information

### Author ORCIDs

Andrei Mironov https://orcid.org/0000-0001-9936-0652

Anton Glushchenko https://orcid.org/0000-0002-3876-3455

Elena Kezlya https://orcid.org/0000-0002-5263-9338

Yevhen Maltsev https://orcid.org/0000-0003-4710-319X

Anton Iurmanov https://orcid.org/0000-0002-0270-8737

Yan Liu https://orcid.org/0000-0001-8556-5040

Maxim Kulikovskiy https://orcid.org/0000-0003-0999-9669

### Data availability

All relevant data can be found within the article text and its Supplementary files.

## Supplementary materials

Supplementary material 1 Alignment of the rbcL and SSU rRNA genes used for phylogenetic analyses in this study Data type txt https://binary.pensoft.net/file/1284138This dataset is made available under the Open Database License (http://opendatacommons.org/licenses/odbl/1.0/). The Open Database License (ODbL) is a license agreement intended to allow users to freely share, modify, and use this Dataset while maintaining this same freedom for others, provided that the original source and author(s) are credited.Author: Andrei Mironov

Supplementary material 2 The Bayesian phylogenetic topology for the rbcL and SSU rRNA genes tree Data type txt https://binary.pensoft.net/file/1284139This dataset is made available under the Open Database License (http://opendatacommons.org/licenses/odbl/1.0/). The Open Database License (ODbL) is a license agreement intended to allow users to freely share, modify, and use this Dataset while maintaining this same freedom for others, provided that the original source and author(s) are credited.Author: Andrei Mironov
